# Arachidonic Acid Metabolism Controls Macrophage Alternative Activation Through Regulating Oxidative Phosphorylation in PPARγ Dependent Manner

**DOI:** 10.3389/fimmu.2021.618501

**Published:** 2021-06-03

**Authors:** Miao Xu, Xiaohong Wang, Yongning Li, Xue Geng, Xudong Jia, Lishi Zhang, Hui Yang

**Affiliations:** ^1^ West China School of Public Health/West China Fourth Hospital and Healthy Food Evaluation Research Center, Sichuan University, Chengdu, China; ^2^ NHC Key Laboratory of Food Safety Risk Assessment, China National Center for Food Safety Risk Assessment, Beijing, China

**Keywords:** macrophage alternative activation, arachidonic acid metabolism, peroxisome proliferator-activated receptor gamma (PPARgamma), oxidative phosphorylation (OXPHOS), prostaglandin E2 (PGE2)

## Abstract

Macrophage polarization is mainly steered by metabolic reprogramming in the tissue microenvironment, thus leading to distinct outcomes of various diseases. However, the role of lipid metabolism in the regulation of macrophage alternative activation is incompletely understood. Using human THP-1 and mouse bone marrow derived macrophage polarization models, we revealed a pivotal role for arachidonic acid metabolism in determining the phenotype of M2 macrophages. We demonstrated that macrophage M2 polarization was inhibited by arachidonic acid, but inversely facilitated by its derived metabolite prostaglandin E2 (PGE2). Furthermore, PPARγ bridges these two seemingly unrelated processes *via* modulating oxidative phosphorylation (OXPHOS). Through inhibiting PPARγ, PGE2 enhanced OXPHOS, resulting in the alternative activation of macrophages, which was counterweighted by the activation of PPARγ. This connection between PGE2 biosynthesis and macrophage M2 polarization also existed in human and mouse esophageal squamous cell carcinoma. Our results highlight the critical role of arachidonic acid and metabolic PGE2 as immune regulators in modulating tissue homeostasis and pathological process.

## Introduction

Metabolic reprogramming is a hallmark of many pathological processes, such as obesity, cancer and cardiovascular diseases. Energy metabolic homeostasis profoundly impacts immune responses in tissue microenvironment (1). When energy is surplus, immune cells reprogram their metabolic pathway to trigger metaflammation (2). Obesity is a prototypical example of how energy metabolic homeostasis affects immunological function. Lipids depositing in various tissues leads to hypoxia and adipocyte stress thus recruits innate immune cells and promotes chronic activation of survival pathway (3). In return, phenotype change of immune cells can also function to regulate system or local metabolic state (4).

Macrophages as one of the prominent components of immune system are versatile. They adopt different polarization states depending on the context of tissue microenvironment. Macrophages sensor, integrate and response to stimulus to achieve metabolic homeostasis through initiating inflammation or insulin action (5). In cancer, metabolic shaping of tumor microenvironment (TME) profoundly impacts the functional responses of immune cells (6). Cancer cells release lactate, glutamine, succinate and α-ketoglutarate (α-KG) and thereby prompt T cells and macrophages to polarize towards immunosuppressive phenotype (7–9). In contrast, metabolic reprogramming of the tumor-associated macrophages (TAMs) inhibits tumor progression by allowing the accumulation of T cell receptor engineered T cells (10). Dysfunction of macrophages contributes to systemic inflammation, thus maintaining the normal state of macrophages is critical for health state (11). Based on functional diversity, macrophages are mainly divided into two phenotypes, classically activated macrophages (M1) and alternatively activated macrophages (M2). Metabolic homeostasis especially within adipose and liver tissues has been found closely related to M2 macrophages, which can promote insulin sensitivity (5, 12). However, the metabolic regulation of macrophage polarization is incompletely understood. Emerging evidences have suggested that macrophages use glucose or fatty acids as fuel sources to attain differential activation (13). How these energy metabolism especially lipid metabolism contribute to macrophage polarization remains unclear.

In this study, we aim to elucidate the mechanism underlying metabolic regulation of macrophage polarization. By using integrated analysis of transcriptomic and lipid metabolomic signatures, we showed that arachidonic acid (AA) metabolism determined the polarization of M2 macrophages. Arachidonic acid and metabolic prostaglandin E2 (PGE2) regulated macrophage polarization induced by IL-4/IL-13. Furthermore, activation of PPARγ by the specific agonist rosiglitazone inhibited the induction of M2 polarization by PGE2. Mechanistically, PGE2 enhanced mitochondrial oxidative phosphorylation (OXPHOS) through suppressing PPARγ, resulting in the M2 polarization of macrophages. Our data suggest arachidonic acid and metabolic PGE2 as critical regulators of macrophage alternative activation.

## Materials and Methods

### Reagents and Antibodies

Arachidonic acid (purity > 98.5%), PGE2 and phorbol 12-myristate 13-acetate (PMA) were obtained from Sigma-Aldrich (St. Louis, MO, USA). Cytokines (IL-4, IL-13 and IFN-γ) were provided by Peprotech (Cranbury, NJ, USA). Fluorescence labeled antibodies and bead-based multiplex LEGENDplex assay were from Biolegend (San Diego, CA, USA). Specific inhibitors were acquired from MedChemExpress (Monmouth Junction, NJ, USA) and Selleckchem (Houston, TX, USA). Information about key reagents was provided in **Supplementary Table S1**.

### Humanized THP-1 Derived Macrophage Polarization Model

Human monocytic THP-1 cells were obtained from American Type Culture Collection (ATCC, VA, USA). THP-1 and THP-1 derived macrophages were maintained in RPMI-1640 medium supplemented with 10% (v/v) fetal bovine serum (FBS), 1% (v/v) penicillin-streptomycin and 0.05 mM 2-mercaptoethanol at a controlled atmosphere with 37°C, 95% relative humidity, 5% CO_2_. To acquire undifferentiated macrophages (M0), THP-1 cells were treated with PMA (25 ng/ml) for 48 h and rest in PMA-free growth medium for 24 h. Interferon-gamma (IFN-γ, 25 ng/ml) and lipopolysaccharide (LPS, 100 ng/ml) were added into M0 for an extra 24 h to obtain M1; Interleukin-4 (IL-4, 20 ng/ml) and Interleukin-13 (IL-13, 20 ng/ml) were added for M2 macrophages. Cells were treated with chemicals as figure captions indicated during the induction of polarization. Anti-CCR7 and anti-CD209 fluorescence labeled antibodies were used to validate M1/M2 macrophages *via* immunofluorescence staining and high content imaging system (HCI, ImageXpress Micro Confocal, Molecular Device, LLC, CA, USA). Cell supernatants were collected for further analysis. The characteristics of M0, M1 and M2 macrophages were validated by morphology, surface markers, gene transcription and functional cytokines (**Supplementary Figures S1**, **S2**).

### Mice Bone Marrow Derived Macrophage (BMDM) Polarization Model

Wild type C57BL/6 mice were obtained from Beijing Vital River Laboratory and C57BL/6 PPARγ^loxP^ mice (*Pparg* ^tm2Rev/J^) were obtained from the Jackson laboratory. Monocyte-specific PPARγ deletion mice (*Pparg* ^−/−ΔMono^) were generated by intercrossing *Pparg* ^tm2Rev/J^ with Lyz2^cre^ mice. Tibias and femurs were isolated from 12-week-old mice. Bone marrow medium (BMM) were prepared by adding M-CSF (10 ng/ml) into DMEM complete medium supplemented with 10% (v/v) FBS and 1% (v/v) penicillin/streptomycin. Bone marrow cells were collected by flushing tibias and femurs with BMM. Next, cells were removed for debris or any remnants with strainers, centrifuged, re-suspended in BMM and cultured for 7 days. BMM was refreshed on day 3, day 5. On day 7, cells were collected and validated through flow cytometry. IFN-γ (25 ng/ml) and LPS (100 ng/ml) were used for M1 polarization. IL-4 (10 ng/ml) and IL-13 (10 ng/ml) were used for M2 polarization. After 48 h, macrophages markers (CD206 for M2, CD69 for M1) were detected by HCI.

### Esophageal Squamous Cancer Carcinoma (ESCC) Mice Model

ESCC mice was established as previously described (14). Briefly, N-Nitrosodimethylamine (NMBA) was administered to C57BL/6 mice by gavage at the dose of 0.25 mg/kg BW, twice a week for 5 weeks. The control group (CT) was given the solvent carboxyl methyl cellulose [CMC, 1% (v/v)] with equivalent volume. All mice were housed in controlled atmosphere with 12 h/12 h light/dark cycle and fed standard chow diets. After gavage, mice were maintained for extra 20 weeks. At the endpoint of experiment, mice were sacrificed and forestomachs were collected and flash frozen in liquid nitrogen for RNA-seq. Transcriptomics data are available in GEO database (http://www.ncbi.nlm.nih.gov/geo/) under the accession number GSE134067.

### Live-Cell High Content Imaging

For surface marker staining, cells in black wall 96-well-plate were washed with PBS and blocked with FcR blocking buffer (FcX block, Biolegend, San Diego, CA, USA) at room temperature for 10 min. Afterwards, cells were incubated with antibodies and Hoechst 33342 in cell staining buffer for 30 min at room temperature. Next, PBS was used for washing and FluoroBrite™ DMEM (Gibco, Grand Island, NY, USA) was used for reducing background fluorescence. For intracellular protein staining, cells were fixed with fix/perm buffer (BD Bioscience) for 30 min and washed with Perm/Wash buffer (BD Bioscience) for twice. Then cells were incubated with primary antibodies at room temperature for 30 min. After washing with Perm/Wash buffer, cells were incubated with fluorescence conjugated secondary antibodies for 30 min. Finally, cells were counterstained for nucleic with Hoechst 33342 and subjected to analysis with ImageXpress software (Molecular Device, LLC, San Jose, CA, USA).

### RNA-Sequencing

Total RNA was extracted from cells or tissues with RNeasy kit (QIAGEN) or TRIzol. RNA quality and quantity was detected with NanoDrop and Agilent 2100 Bioanalyzer. After that, mRNA was enriched with Oligo (dT) magnetic beads and broke into short fragments for cDNA synthesis. The cleaved RNA fragments were reversely transcribed into first strand cDNA using random hexamers, following by second strand cDNA synthesis using DNA Polymerase I and RNase H. The double-stranded cDNA was purified, added A tail and connected with a sequencing adapter. Then, PCR amplification was performed on ABI StepOnePlus Real-Time PCR System and the constructed sequencing library was sequenced at Illumina HiSeq. Raw RNA sequencing data is available through the National Center for Biotechnology Information Gene Expression Omnibus (NCBI–GEO) database (http://www.ncbi.nlm.nih.gov/geo/) under the accession number GSE159112, GSE159120.

Raw data was filtered and clean reads were aligned with reference genome (hg19) using HISAT. Total mapped reads of all samples are higher than 95%. Reads were reconstructed into transcripts and their abundance was estimated and expressed as Fragments per kilo base per million mapped reads (FPKM). DEseq2 was used to determine differentially expressed genes (DEGs).Fold change≥ 2 or ≤ 0.5 and adjust *p* value ≤ 0.05 were set for DEGs. Kyoto Encyclopedia of Genes and Genomes (KEGG) pathway analysis was performed with R phyper. Heatmaps were generated with online tools (http://www.ehbio.com/ImageGP/index.php/Home/Index).

### Gene Set Enrichment Analysis (GSEA)

All FPKM values of identified genes from RNA-sequencing were input into GSEA software 4.0.3 for enrichment analysis (15). Data were normalized first and then a ranked gene list were generated. Database was downloaded from Molecular Signatures Database (MSigDB) gene sets (http://software.broadinstitute.org/gsea/index.jsp).

### Lipid Metabolomics Analysis

M0/M1/M2 macrophages were collected and immediately stored at liquid nitrogen until analysis. Samples were thawed on ice and added 800 μl pre-chilled dichloromethane/methanol (3: 1) buffer, then precipitated in refrigerator at -20°C for 2 h. Then samples were centrifuged at 25,000 g, 4°C for 15 min. The supernatants (650 µL/each) were transferred to new tubes and centrifuged again. Then the supernatants (600 µL/each) were frozen-dry and reconstituted by lipid reconstituted solution (isopropanol: acetonitrile: water = 2:1:1, 600 µL/each). After centrifuging, the supernatants (60 µL/each) were detected on the LC-MS system. Quality control (QC) was obtained by mixing the supernatants from 3 samples (20 µL/each) and detected under same condition.

Raw data from mass spectrometer were firstly preprocessed (noise filtering, peak matching and extraction), and corrected based on the quality control-based robust LOESS signal correction (QC-RSC). Human Metabolome Database (HMDB) and LipidMaps database were used for peak alignment. Secondly, multivariate analysis principal component analysis (PCA) and partial least squares-discriminant analysis (PLS-DA) were introduced to test for difference. Metabolites with fold change ≥ 1.2 or ≤ 0.8333 and q-value < 0.05 were selected as differential metabolites. Finally, the differential metabolites identification were performed with Progenesis QI (version 2.2) software. Pathway analysis was based on KEGG database.

### Metabolite Set Enrichment Analysis (MSEA) and Joint Pathway Analysis

Differential lipid metabolites from positive or negative ion mode were input for Metabolite Set Enrichment Analysis with online tools (MetaboAnalyst, https://www.metaboanalyst.ca) as previous study introduced (16).

Differential lipid metabolites and DEGs from RNA-sequencing were input simultaneously to conduct joint pathway analysis on MetaboAnalyst. Integrated metabolic pathway database from current KEGG version was chosen for enrichment. Parameter listed as follow: hyper geometric test for enrichment analysis, closeness centrality for topology measure and overall combine *p* value for integration method.

### Macrophages Cytokines Determination

After induction of polarization, culture medium was refreshed and 24 h later, cell supernatants were collected for cytokines determination. The concentration of cytokines was measured with LEGENDplex™ Human macrophage panel using flow cytometry following manufacturer’s instruction. Data were analyzed with Legendplex software (v8.0).

### Correlation Analysis

The mRNA expression data used for correlation analysis in this study is available in the Genomic Data Commons (https://portal.gdc.cancer.gov/). Briefly, a total of 90 cases of ESCC were included, and clinical characteristics had been described in previous study (17). FPKM values were log transformed (log_2_(X+1), X= raw FPKM) for analysis. Pearson correlation coefficients and liner regression were analyzed with Graphpad Prism 6.

### Statistical Analysis

All quantitative experimental values were presented as mean ± SEM. Data were processed and visualized with Graphpad Prism 6. Unpaired t test or ANOVA analysis were applied to determine statistical significance within different treatments. P < 0.05 was set for significance.

## Results

### Macrophage M2 Polarization Is Tightly Associated with Lipid Metabolism

**Figure 1:**
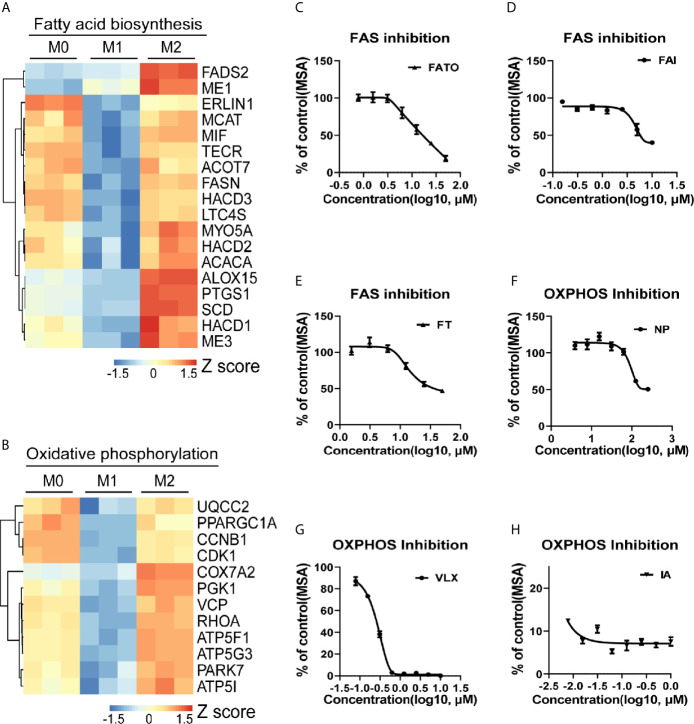
Macrophage M2 polarization is tightly associated with lipid metabolism. **(A, B)** Gene expression in THP-1 derived differentially activated macrophages related to fatty acid biosynthesis (FAS) or oxidative phosphorylation (OXPHOS), respectively. **(C–E)** CD209 expression curves of THP-1 derived M2 macrophages *via* high content imaging (HCI) with specific FAS inhibitors treated as indicated for 48 h. **(F–H)** CD209 expression curves of THP-1 derived M2 macrophages *via* HCI with specific OXPHOS inhibitors treated as indicated for 48 h. Error bars represent the mean ± SEM from 3 biological replicates. MSA, mean stain area.

To investigate the role of lipid metabolism in the regulation of macrophage polarization, we analyzed the transcriptomic changes with THP-1 derived macrophage polarization model. M1/M2 macrophages showed divergent features of energy metabolism when compared with M0 macrophages (**Figure 1** and **Supplementary Figure S3**). The expression of genes controlling fatty acid biosynthesis (FAS) (**Figure 1A**) and OXPHOS (**Figure 1B**) was particularly enhanced in M2 macrophages. To validate whether these pathways contribute to M2 polarization, cells were treated with series of inhibitors in the induction of M2 polarization. FAS inhibition induced by FASN-IN-4 tosylate (FAI), Fatostatin (FATO), FT113 (FT) dose-dependently decreased CD209 expression (**Figures 1C–E**), suggesting that M2 polarization could be promoted by the activation of FAS pathway. Similarly, blockade of OXPHOS by 3-Nitropropanoic acid (NP), VLX600 (VLX) and IACS-10759 (IA) (**Figures 1F–H**) also attenuated macrophage M2 polarization in a dose-dependent manner. Other processes associated with lipid utilization including lipolysis, fatty acid transport (FAT) and fatty acid oxidation (FAO) did not show featured alternation during the M2 polarization (**Supplementary Figures S3A–C**). However, inhibition of these processes also affected M2 polarization (**Supplementary Figures S3D–F**). Inhibition of FAT and FAO significantly suppressed M2 polarization (**Supplementary Figures S3D, E**). This is in line with the results from blockade of FAS and OXPHOS. In contrast, inhibition of lipolysis dramatically promoted macrophage M2 polarization (**Supplementary Figure S3F**). These data suggested that fatty acid biosynthesis and utilization were crucial for macrophage M2 polarization.

### Arachidonic Acid Metabolism Is Enhanced in M2 Macrophages

**Figure 2:**
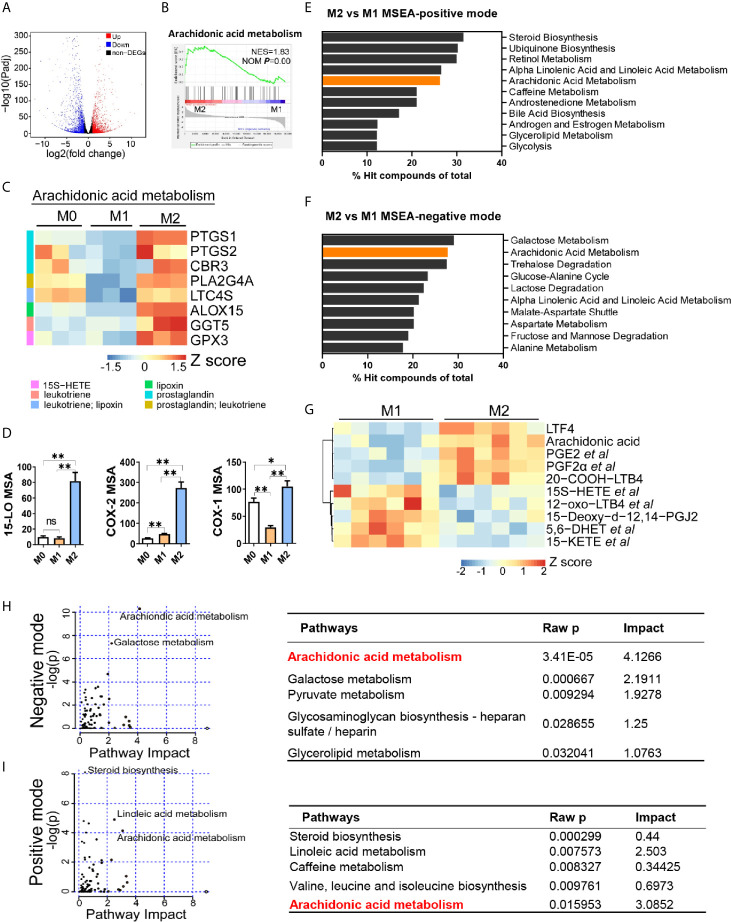
Arachidonic acid metabolism is enhanced in M2 macrophages. **(A)** Differentially expressed genes (DEGs) in M2 macrophages; fold changes are in comparison with M1 macrophages. Genes with fold change ≥ 2 or ≤ 0.5 and Padj ≤ 0.05 were seen as DEGs. **(B)** Enrichment plot for arachidonic acid metabolism in THP-1 derived M2 macrophages from Gene Set Enrichment Analysis (GSEA). **(C)** Heatmap of DEGs matching “arachidonic acid metabolism” expression signature according to KEGG Pathway Analysis of RNA-sequencing data from M0, M1, M2 macrophages with three biological replicates. **(D)** Protein expression of key metabolic enzymes for arachidonic acid *via* HCI in M0, M1, M2 macrophages. *P < 0.05; **P < 0.01. Error bars represent the mean ± SEM from three biological replicates. **(E, F)** Top 10 pathways of positive or negative ion mode from Metabolites Set Enrichment Analysis (MSEA). **(G)** Heatmap of differentially expressed metabolites matching “arachidonic acid metabolism” expression signature according to KEGG Pathway Analysis of lipidomics data from M1,M2 macrophages with six biological replicates. **(H, I)** Enrichment pathways from integrated transcriptomics and lipidomics data by Joint Pathway Analysis. Left panel: Enrichment plots. Right panel: corresponding information for left plots.

Next, we aimed to identify the key lipid metabolic regulator in M2 polarization by the transcriptomic and metabolomic analysis. Firstly, among all DEGs between M1 and M2 macrophages, 1645 genes were up-regulated in M2 macrophages (**Figure 2A**). Analysis of the expression profiles against the hallmark gene sets available from MSigDB suggested an enrichment of arachidonic acid metabolism in M2 macrophages (**Figure 2B**). Expression level of genes associated with arachidonic acid metabolism were significantly higher in M2 macrophages (**Figure 2C**). Elevated expression of these genes was largely related to prostaglandins and leukotrienes production (**Figure 2C**). In accordance, the expression of key metabolic enzymes that utilize arachidonic acid as a substrate for the synthesis of eicosanoids, including 15-lipoxygenase (15-LO, encoded by ALOX15) and cyclooxygenases (COX-1/COX-2, encoded by PTGS1/PTGS2) were significantly elevated in M2 macrophages (**Figure 2D**).

To decipher the lipid metabolic signature for macrophage polarization, we further analyzed metabolomic difference between M1 and M2 macrophages. PCA and PLS-DA analysis revealed metabolic disparity of M1 and M2 macrophages (**Supplementary Figures S4A, B**). A total of 3652 and 2328 differential ions (identified as 808 and 510 differential metabolites) were obtained in positive and negative mode, respectively (**Supplementary Figure S4C**). We next performed MSEA to these differential metabolites. Top 10 enriched pathways showed that arachidonic acid metabolism was the only two pathways that included in both modes, ranking 5^th^ and 2^nd^ respectively (**Figures 2E, F**). Another pathway, alpha linoleic acid and linoleic acid metabolism, was also included in top 10 pathways (**Figures 2E, F**). This is possibly due to that it shares some common enzymes with arachidonic acid metabolism. Next, we compared differential metabolites associated with arachidonic acid metabolism. Metabolites profiles of M1 and M2 were significantly different (**Figure 2G**, **Supplementary Table S2**). Arachidonic acid, and prostaglandins (PGE2, PGF2α et al.) and leukotrienes (LTF4, 20-COOH-LTB4) were enriched in M2 macrophages. Integrated analysis of transcriptomics and metabolomics demonstrated that arachidonic acid metabolism was the most remarkable pathway with highest pathway impact in both modes (**Figures 2H, I**). Other metabolic pathways such as linoleic acid pathway and galactose metabolism were also significantly changed but with lower pathway impact (**Figures 2H, I**). Together, these data revealed that arachidonic acid metabolism was the most remarkable lipid metabolism disparity between M1 and M2 macrophages. Enhanced arachidonic acid metabolism could be a hallmark of M2 macrophages.

### Arachidonic Acid and PGE2 Inversely Regulate M2 Polarization

**Figure 3:**
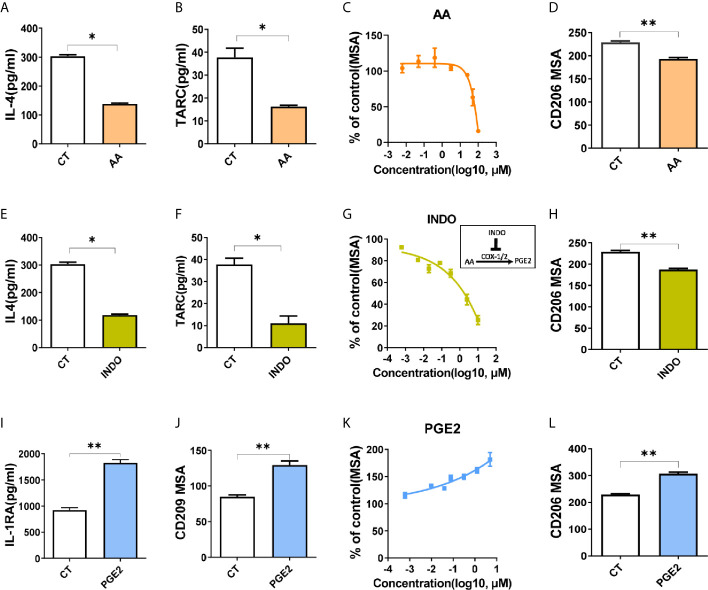
Arachidonic acid and PGE2 inversely regulate M2 polarization. **(A, B, E, F, I)** Cytokines of THP-1 derived M2 macrophages treated with compounds for 48hours during polarization as indicated. **(C, G, K)** CD209 expression curves of THP-1 derived M2 macrophages with treatment as indicated *via* HCI **(D, H, L)** Analysis of CD206 expression of BMDM derived M2 macrophages *via* HCI with treatments as indicated. **(J)** Analysis of CD209 for THP-1 derived M2 macrophages treated as indicated during polarization *via* HCI. CT represents corresponding solvent control. Arachidonic acid (AA, 50 μM), indomethacin (INDO, 10 μM), prostaglandin E2 (PGE2, 10 μM). Error bars represent the mean ± SEM. Data presented are from three biological replicates. *P < 0.05; **P < 0.01.

Next, we aimed to investigate the impact of arachidonic acid metabolism on M2 polarization *in vitro*. By treating cells with arachidonic acid during polarization, we found that both surface markers (CD209 for THP-1 model, CD206 for BMDM model) and functional cytokines (IL-4, TARC) had been decreased by arachidonic acid, indicating that macrophage M2 polarization was suppressed (**Figures 3A–D**). In addition, the key enzymes associated with arachidonic acid metabolism are generally constitutively expressed and determine what eicosanoids a cell can synthesize. Our data revealed relative expression of lipoxygenases and cyclooxygenases in M2 macrophages (**Figure 2**), thus we tested how these enzymes link to M2 polarization. We found that inhibition of lipoxygenases (by PD146176 and MK886) decreased M2 polarization in a dose-dependent manner (**Supplementary Figures S5A, B**). Consistently, inhibition of cyclooxygenases by indomethacin (INDO) decreased M2 polarization, which was indicated by lower expression of IL-4, TARC and CD209, CD206 (**Figures 3E–H**). This suggested that metabolites of arachidonic acid may favor M2 polarization. Thus we assessed the expression of markers in the presence or absence of corresponding arachidonic acid metabolites. We tested several lipoxygenases related metabolites lipoxin A4 (LXA4), 15-hydroxyeicosatetraenoic acid (15S-HETE) and leukotriene B4 (LTB4). Neither of them affected macrophage M2 polarization in THP-1 model or BMDM model (**Supplementary Figures S5C, D**). However, the presence of an cyclooxygenases associated metabolite, PGE2, significantly promoted M2 polarization, as increasingly expressed M2 markers (IL-1RA, CD209, CD206) suggested (**Figures 3I–L**). In addition, all these metabolites inhibited M1 polarization (indicated by CCR7 expression) in THP-1 model (**Supplementary Figure S5E**) while only PGE2 inhibited M1 polarization (indicated by CD69 expression) in BMDM model (**Supplementary Figure S5F**), suggesting that PGE2 may determine the polarization of M1/M2 polarization. Collectively, these data indicated a critical role for arachidonic acid and its metabolic PGE2 in optimal M2 polarization of macrophages induced by IL-4/IL-13.

### PGE2 Facilitates Macrophage M2 Polarization Through PPARγ Suppression

**Figure 4:**
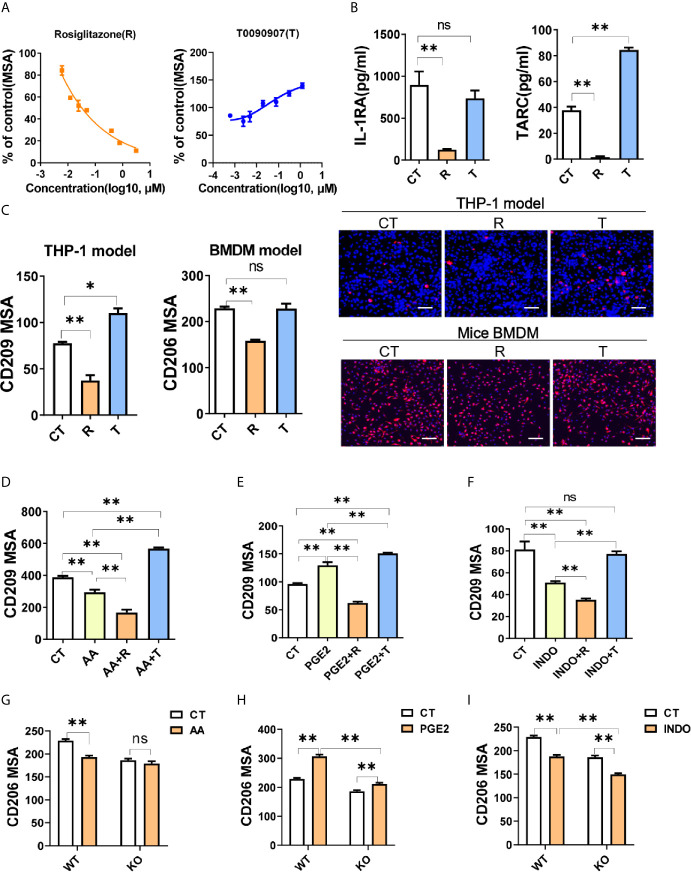
PGE2 facilitates macrophage M2 polarization through PPARγ suppression. **(A)** CD209 expression curves of THP-1 derived M2 macrophages with treatments as indicated. **(B)** Cytokines of THP-1 derived M2 macrophages with treatment as indicated. **(C)** Protein expression of M2 markers (CD209 or CD206) from THP-1 or BMDM derived M2 macrophages with treatment as indicated. Right panel: representative images. Blue staining: nuclei; Red staining: CD209 or CD206. Scale bar: 100 μm. **(D–F)** Protein expression of CD209 in THP-1 derived M2 macrophages with treatment as indicated. **(G–I)** Protein expression of CD206 for BMDM derived M2 macrophages from wide type (WT) or monocyte specific PPARγ knockout mice (KO) with treatment as indicated. CT represents corresponding solvent control. Rosiglitazone (R, 10 μM), T0070907 (T, 1 μM), AA (50 μM), INDO (10 μM), PGE2 (2 μM). Error bars represent the mean ± SEM from three biological replicates. *P < 0.05; **P < 0.01; ns, no significance.

Previous studies had demonstrated that PPARγ were essential for M2 polarization (5), therefore we investigated the involvement of PPARγ in the molecular mechanism of macrophage polarization induced by arachidonic acid and PGE2. When treated with specific agonist for PPARγ, rosiglitazone (R), M2 marker (CD209) was decreased dose-dependently (**Figure 4A**). In contrast, CD209 was significantly enhanced by the inverse agonist T0070907 (T) dose-dependently (**Figure 4A**), suggesting that human macrophage M2 polarization might be closely associated with PPARγ de-activation. Functional cytokines secreted by M2 macrophages (IL-1RA and TARC) were correspondingly reduced by PPARγ activation and TARC was increased by PPARγ de-activation (**Figure 4B**). In consistent with THP-1 model, PPARγ activation by R inhibited macrophage M2 polarization in BMDM model as well (**Figure 4C**). These data suggested that PPARγ de-activation was critical for M2 polarization.

Since the inhibition effect of arachidonic acid on M2 polarization (**Figures 3A–D**) was similar to R, we presumed that arachidonic acid inhibited macrophage M2 polarization through activating PPARγ. To test this, we examined the polarization effect of arachidonic acid in the presence of T. When PPARγ de-activated by T, arachidonic acid could not inhibit M2 polarization while PPARγ activated by R could enhance the inhibition of arachidonic acid on CD209 expression, suggesting that PPARγ was involved in the effect of arachidonic acid and might be activated by arachidonic acid (**Figure 4D**). At the same time, we questioned whether PGE2 promoted M2 polarization by suppressing PPARγ activation. We found that PPARγ activation totally reversed M2 polarization mediated by PGE2 while PPARγ de-activation further enhanced M2 polarization mediated by PGE2 (**Figure 4E**). In addition, INDO shared a similar response with arachidonic acid when co-treated with R or T, suggesting that PPARγ was involved in the effect of INDO and might be activated by INDO (**Figure 4F**). To formally address the possibility of PPARγ in bridging arachidonic acid and/PGE2 mediated macrophage M2 polarization, we construct BMDM polarization model from wild type (WT, *Pparg* ^+/+ΔMono^) or monocyte specific PPARγ knockout (KO, *Pparg* ^−/−ΔMono^) mice. In WT model, M2 marker CD206 was significantly suppressed by arachidonic acid while increased by PGE2 (**Figures 4G, H**). However, in KO mice, when compared with WT, monocyte specific PPARγ knockout significantly abolished or dampened these effects on M2 polarization (**Figures 4G, H**), indicating that arachidonic acid and/PGE2 regulated M2 polarization in a PPARγ-dependent manner. Intriguingly, the suppression of CD206 by INDO was not abolished but was enhanced by monocyte specific PPARγ knockout, suggesting that INDO might have additional mechanisms besides PPARγ activation in regulating M2 polarization of macrophages (**Figure 4I**). Together, these data supported the proposal of a role for PPARγ in bridging arachidonic acid or PGE2 mediated macrophage M2 polarization.

### PGE2 Enhances OXPHOS Through Suppressing PPARγ in Promotion of Macrophage M2 Polarization

**Figure 5:**
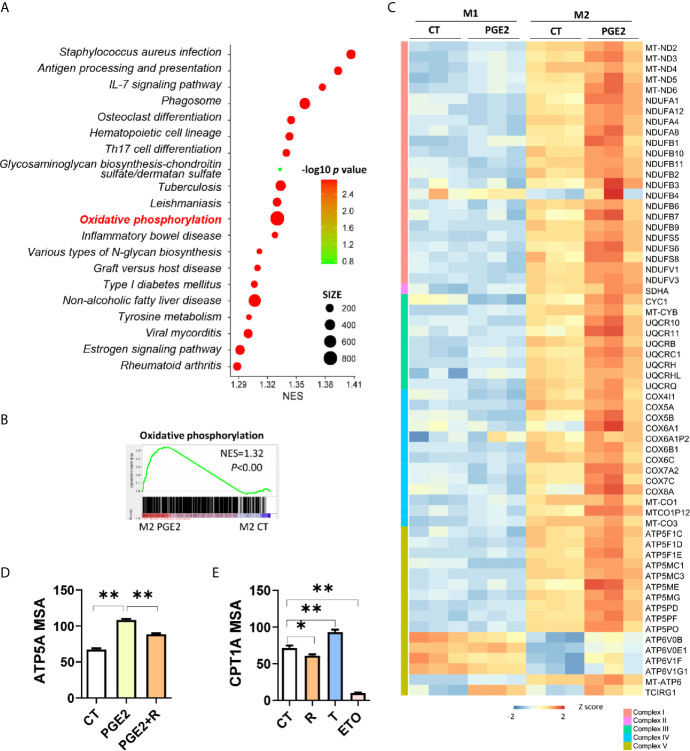
PGE2 enhances OXPHOS through suppressing PPARγ in promotion of macrophage M2 polarization. **(A)** Enrichment pathways [TOP 20, ranked with normalized enrichment score (NES)] in THP-1 derived M2 macrophages with PGE2 treated during polarization. **(B)** Enrichment plot for oxidative phosphorylation in THP-1 derived M2 macrophages with PGE2 treated during polarization from GSEA analysis. **(C)** Heatmap for representative DEGs matching “oxidative phosphorylation” in PGE2 treated M1 or M2 macrophages. **(D)** Protein expression of ATP5A with treatment during polarization as indicated and detected *via* HCI. **(E)** Protein expression of CPT1A with treatment during polarization as indicated. PGE2 (2 μM), R (10 μM), T (1 μM), ETO (Etomoxir, 100 μM, serve as positive control for CPT1A inhibition). *P < 0.05; **P < 0.01.

On the basis of OXPHOS was enhanced in M2 macrophages (**Figure 1B**) and FAO fuels OXPHOS with acetyl-CoA (18), we proposed that the polarization effects of PGE2 or AA might be attributed to these two processes. To test this, we conducted transcriptomic analysis of PGE2 treated macrophages in the induction of M2 polarization. Firstly, we explored the top 20 enriched pathways (ranked by normalized enrichment score, NES) by GSEA (**Figure 5A**). When considering false discovery rate q-value (FDR q value, usually no more than 0.25 was acceptable), the 11^th^ pathway, *oxidative phosphorylation*, was the most significantly enriched pathway with highest NES (**Figure 5A** and **Supplementary Table S3**). The enrichment plot of OXPHOS suggests that PGE2 up-regulated OXPHOS remarkably in the induction of M2 polarization (**Figure 5B**). Further comparison on the OXPHOS associated DEGs demonstrated that genes related to mitochondria respiratory complex I (NDUFA8 et al.), II (SDHA), III (UQCRH et al.), IV(COX7A2 et al.), V (ATP5MG et al.) were exclusively increased in the induction M2 but not M1 polarization (**Figure 5C**). This was consistent with the enhancement of OXPHOS in M2 macrophages (**Figure 1C**). To explore whether PPARγ was involved in the enhancement of OXPHOS by PGE2, we tried to block this effect by PPARγ activation. As expected, PGE2 dramatically increased the expression of ATP5A, one subunit of ATP synthase (complex V), while PPARγ activation weakened this effect significantly, suggesting that PPARγ was involved in the effect of PGE2 on OXPHOS (**Figure 5D**). In addition, given that FAO can serve as a replenishment pathway for OXPHOS and PPARγ is a key regulator for FAO, we next determine the involvement of PPARγ on FAO. CPT1A expression, a key enzyme for FAO, was significantly inhibited by PPARγ activation while was promoted by PPARγ de-activation (**Figure 5E**). This indicated that PPARγ activation inhibited FAO, thus might weaken OXPHOS. This finding was consist with a previous study that demonstrated the inhibition of OXPHOS by PPARγ activation (19). Collectively, these data revealed that PGE2 enhanced OXPHOS during M2 polarization and PPARγ de-activation was involved in this process.

### Arachidonic Acid Metabolism Is Correlated With M2 Polarization in Tumor Microenvironment

**Figure 6:**
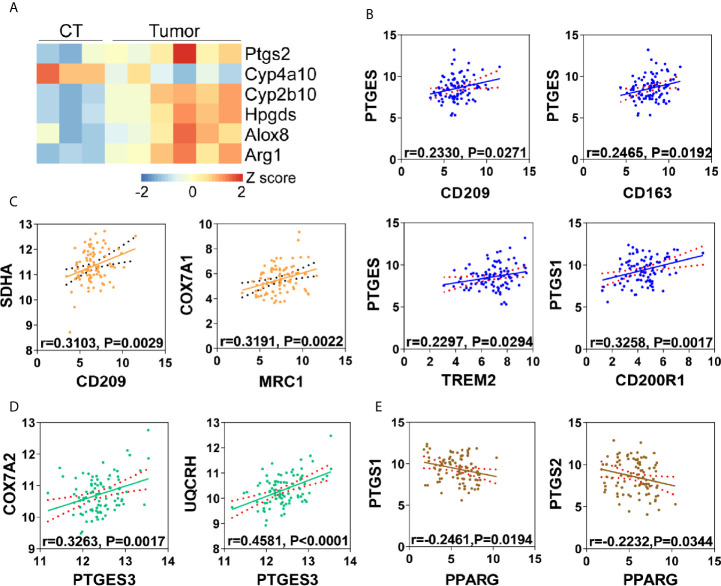
Arachidonic acid metabolism is correlated with M2 polarization in tumor microenvironment. **(A)** Heatmap of differentially expressed genes matching “arachidonic acid metabolism” from mice ESCC or corresponding control tissues (CT). Arg1 was used as M2 macrophages marker. **(B)** Correlation analysis of PGE2 biosynthesis (PTGES, PTGS1) and M2 macrophages (CD209, CD163, CD200R1, TREM2) in human ESCC from TCGA database. **(C)** Correlation analysis of OXPHOS (COX7A1, SDHA) and M2 macrophages (CD209, MRC1) in human ESCC from TCGA database. **(D)** Correlation analysis of oxidative phosphorylation (COX7A2, UQCRH) and PGE2 biosynthesis (PTGES3) in human ESCC from TCGA database. **(E)** Correlation analysis of PPARG and PGE2 biosynthesis (PTGS1, PTGS2) in human ESCC from TCGA database.

The metabolic crosstalk between cancer cells and macrophages suggested that nutrients availability may play a role in immunosuppressive tumor microenvironment (20, 21). M2 type tumor associated macrophages (M2-TAMs) formation have been seen as results of tumor cell “re-education” (22). This suggests that tumor cells derived metabolites may have a role for M2-TAMs formation. In our previous study, we have validated M2-TAMs infiltration in esophageal carcinogenesis (17), thus we questioned whether arachidonic acid metabolism facilitated M2-TAMs polarization in esophageal cancer. Unsurprisingly, in mice ESCC, comparing with non-tumor tissue, several key metabolic genes in arachidonic acid metabolism *(Ptgs2, Cyp4a10, Cyp2b10, Hpgds *and* Alox8)* were up-regulated in tumor tissues and M2 macrophages marker Arg1 was also increased in tumor tissues (**Figure 6A**), indicating a correlation between arachidonic acid metabolism and M2-TAMs formation. Simultaneously, using transcriptomics data from human ESCC, we also observed that many markers for M2 macrophages such as MRC1, CD209, CD163 and TREM2 were positively correlated to PGE2 biosynthesis (suggested by PTGES and PTGS1) of arachidonic acid metabolism (**Figure 6B**). Thus we next check the correlation between FAO/OXPHOS and M2-TAMs. Several FAO or OXPHOS associated genes (PPARGC1A, COX7A1, SDHA) positively correlated to markers of M2-TAMs (CD200R1, MRC1, CD209, CD163) (**Figure 6C** and **Supplementary Figure S6)**. This suggested the existence of OXPHOS related M2-TAMs formation. Consistently, PGE2 biosynthesis (suggested by PTGES3) and OXPHOS (suggested by UQCRH, COX7A2) were also positively correlated (**Figure 6D**). This was consistent with our *in vitro* observation and support our hypothesis (arachidonic acid metabolism facilitates M2-TAMs polarization in esophageal cancer) well. In addition, by calculating the correlation between PTGS1/PTGS2 and PPARG, we found that PGE2 biosynthesis was negatively correlated to PPARG (**Figure 6E**), which indirectly supported that PGE2 suppressed PPARG. Together, these findings suggest that arachidonic acid metabolism might make contribution to M2-TAMs formation *via* PPARγ-OXPHOS modulation, thus promote tumor progression.

## Discussion

**Figure 7:**
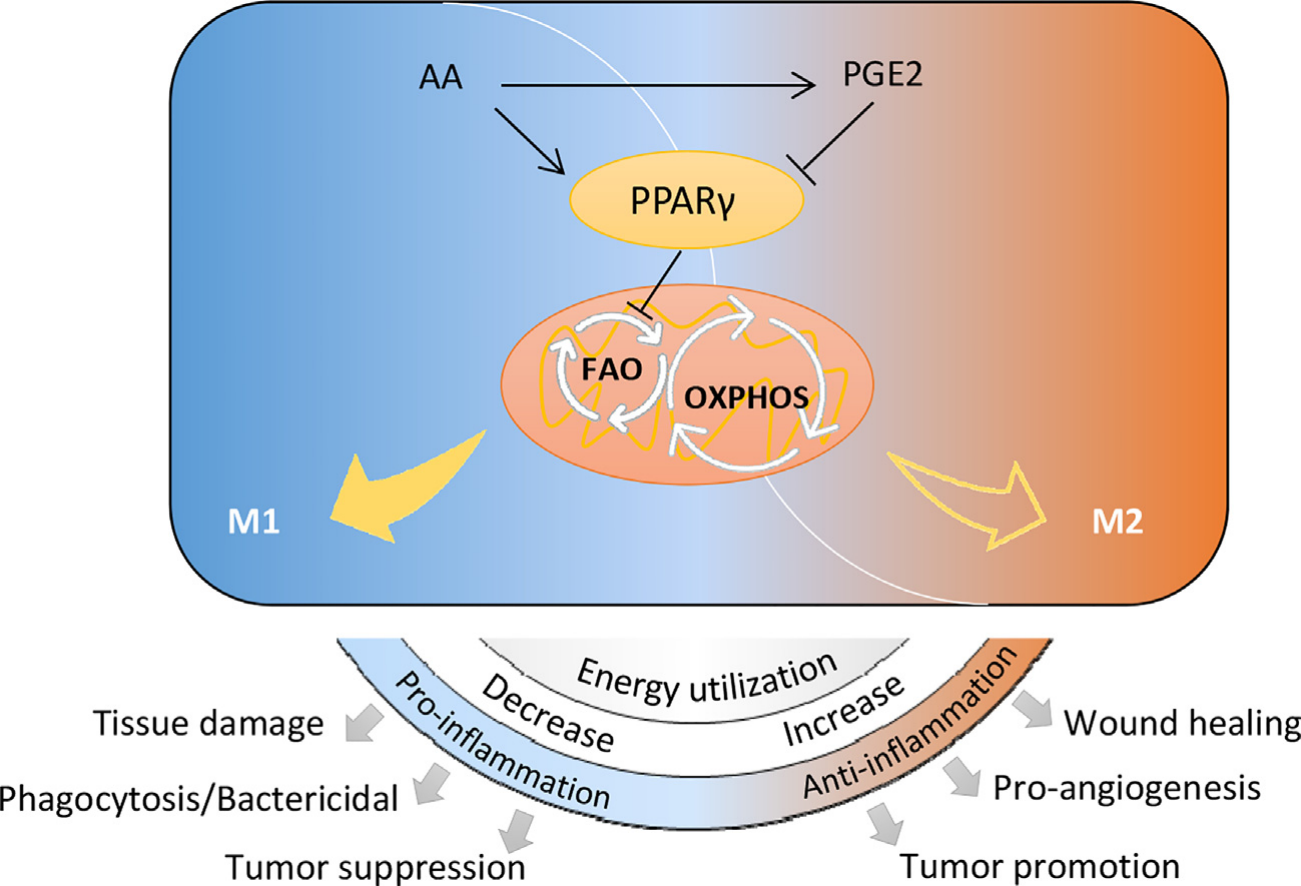
Schematic diagram of arachidonic acid metabolism in controlling macrophage polarization and biological processes.

Distinct metabolic characteristics help macrophages with particular function during phenotype polarization (13). Lipid mediators are key fatty acid metabolites involved in this process (13), serving as important signals. In the present study, we demonstrate that arachidonic acid metabolism is up-regulated in the induction of macrophage M2 polarization. Arachidonic acid inhibits IL-4/IL-13 stimulated M2 polarization of macrophages. PGE2, an essential metabolite generated from arachidonic acid metabolism, promotes macrophage M2 polarization through inhibiting PPARγ. Contrary to PGE2, inhibition of arachidonic acid metabolism suppresses M2 macrophage polarization. Our data elucidates a previously unappreciated mechanism of Arachidonic acid metabolic PGE2 to regulate macrophage alternative activation through inhibiting PPARγ. This inhibition effect facilitates OXPHOS by enhancing FAO pathway. The newly uncovered connection between arachidonic acid metabolism and macrophage alternative activation is briefly outlined in **Figure 7**. This metabolic regulation of macrophage polarization will affect many physiological and pathological processes eventually.

Metabolism intricately links to immune homeostasis, which largely reflects by polarization of immune cells including macrophages towards differential phenotypes. Like any physiological process, polarization of immune cells requires energy as well as the availability of nutrients, metabolites and oxygen (23). Macrophages in a nutrient deprivation or hypoxia microenvironment acquire distinct phenotypes with those in perivascular areas (24), suggesting an essential role of oxidative metabolism of nutrients. Mitochondrial OXPHOS has been widely accepted as characteristic and necessity for M2 macrophages (25, 26). This biological process is essential in M2 macrophages for ATP and biosynthetic output. Roisin et al. has reviewed how oxidative metabolism controls immune cell function (27). Inhibition of OXPHOS by reducing substrates or inhibiting mitochondrial complex had been found to suppress M2-related genes (Arg1, Mrc1) and surface marker (CD206) (26, 28). In line with these observations, we found that OXPHOS directly control macrophage alternative activation (M2 polarization).

Mitochondria utilizes pyruvate from glucose metabolism, α-KG from glutamine metabolism as well as acetyl-CoA from FAO to feed Krebs cycle and drive OXPHOS. It has been known that in M2 macrophages, FAO fuels OXPHOS thus provides a crucial energy source for M2 polarization (25, 26). Pharmaceutically blockade of FAO diminishes immune function of M2 macrophages or dampens M2 polarization (25) and favors M2-to-M1 repolarization (29), suggesting an essential role of FAO in macrophage M2 polarization. Our data also verify its contribution on M2 polarization by FAO inhibitor, which significantly decreased CD209 dose-dependently. Due to PPARs, especially PPARγ, have been extensively investigated as essential nuclear receptors for macrophage M2 polarization and in lL-4 stimulated M2 polarization, PPARγ and PGC1β are well known regulators of FAO (26, 30), we speculated that PPARγ-regulated FAO might have a role in M2 polarization. Interestingly, our data revealed that PPARγ negatively regulated CPT1A (an important enzyme for FAO) during M2 polarization, suggesting an inhibition of FAO by PPARγ. Collectively, these data demonstrate that FAO regulated by PPARγ are involved in M2 polarization.

In our study, PGE2 was found to dramatically promote M2 polarization and enhance OXPHOS during this process, revealing a possibility that PGE2 promotes macrophage M2 polarization through enhancing OXPHOS. On the basis of previous studies, PPARγ regulated FAO may be involved in this process (31). Besides, our results show that PGE2 directly suppress PPARγ expression and transcription activity (**Supplementary Figure S7**). Therefore, it’s conceivable that inhibition of PPARγ to favor FAO can enhance OXPHOS and lead to the promotion effect of PGE2 in M2 polarization. Consistently, PPARγ activation might be a potential mechanism of arachidonic acid in inhibiting M2 polarization. In addition, PGE2-EP4 signaling has been reviewed as a possible mechanism of M2 polarization (32). However, we found that PGE2 promoted M2 polarization even though EP4 was blocked (**Supplementary Figure S8**). This suggests the existence of EP4-independent mechanisms under PGE2 mediated M2 polarization. Therefore, we believe that this PPARγ dependent mechanism is the main contributor in PGE2 mediated M2 polarization.

As for the role of lipolysis on macrophage M2 polarization, previously studies reported that lipolysis inhibition suppressed M2 polarization due to reduced FAO (25) or blocked PGE2 biosynthesis (33). However, the target of JZL184, monoacylglycerol lipase (MAGL), can metabolize 2-arachidonoylglycerol into arachidonic acid (34), thus JZL184 can decrease arachidonic acid intracellular biosynthesis. Based on our data that arachidonic acid inhibited M2 polarization through activating PPARγ, the promotion effect of JZL184 on M2 polarization might be attributed to arachidonic acid reduction. In addition, it should be note that large amount of fatty acids and lipid mediators are pan-agonist for PPARs, thus PPARα and PPARδ may be also involved in arachidonic acid metabolism regulated M2 polarization. We have found that PPARα was not influenced by arachidonic acid or PGE2 (**Supplementary Figure S9A**) while PPAR response element (PPRE) and CD36 could be activated by PGE2 (**Supplementary Figures S9B, C**), suggesting the involvement of PPARs other than PPARα. The role of PPARδ might be also involved in PGE2 mediated macrophage polarization (35, 36). There is a need to clearly compare polarization effects between PPARs and their contribution to PGE2 mediated macrophage polarization. Besides, many other metabolites could also be produced by arachidonic acid metabolism, the polarization effect of this pathway may be more complex beyond our data suggest. Further investigations are needed to clearly clarify our findings.

The identification of PGE2 as a key player for macrophage M2 polarization adds a metabolic explanation for how tumor polarize infiltrated macrophages towards an immunosuppressive M2 type. Previous studies have demonstrated that PGE2 promotes TAMs formation in glioblastoma (33), colorectal cancer (35), ovarian cancer (37), neuroblastoma (38) and prostate cancer (39). Based on the accumulation of macrophages (40) and differential expression of COX-2 in ESCC (41), it could be inferred that above correlation may also exist in the TME of ESCC. Our data supported this opinion and also suggested that besides tumor cell-derived PGE2, macrophage-derived PGE2 may also make contribution to M2-TAMs polarization through PPARγ-OXPHOS pathway. In addition, this correlation may generally exist in many other physiological/pathological conditions. Some *in vivo* experiments have revealed that PGE2 administration promoted M2 macrophage polarization. In a xenograft mouse model of colorectal cancer, PGE2 (17.6 μg/kg/d) treatment increased CD206^+^ M2 macrophages in TME (42). However, in a mice asthma model, although PGE2 (0.4 mg/kg) administration decreased Ym-1 (M2 marker), it did not affect CD206 expression (43). This uncertain polarization effect is possibly due to the sophisticated responses of various cell types that can be affected by free PGE2 in the lung. These findings indicate that metabolic production of PGE2 may serve as a potential target for the prevention of many macrophages associated diseases including cancers. Reduction of PGE2 by pharmacological inhibition of COX-1/COX-2 may benefit cancer prevention. INDO, a non-specific COX-1/COX-2 inhibitor, has shown chemo-preventive and chemotherapeutic efficacy on colorectal cancer (44).

In summary, we identified that arachidonic acid metabolism notably impact macrophage M2 polarization induced by IL-4/IL-13 through regulating PPARγ and OXPHOS. As one of the key metabolite of arachidonic acid, PGE2 plays a crucial role in promoting macrophage polarization by the inhibition of PPARγ and enhancement of OXPHOS. Our finding renews the current understanding about the functions of arachidonic acid and metabolic PGE2 as immune regulators.

## Data Availability Statement

The datasets presented in this study can be found in online repositories. The names of the repository/repositories and accession number(s) can be found in the article/**Supplementary Material**.

## Ethics Statement

The animal study was reviewed and approved by Institutional Animal Care and Use Committee of China National Center for Food Safety Risk Assessment.

## Author Contributions

MX: Writing-Original Draft preparation, Methodology, formal analysis, investigation. XW: Validation, investigation. YL & XG: investigation. XJ: Supervision, Funding acquisition LZ: Supervision, Project administration. HY: Conceptualization, Data Curation, Resources, Writing - Review & Editing. All authors contributed to the article and approved the submitted version.

## Funding

This study was funded by National Key Research and Development Program of China (No. 2018YFC1603102) and National Natural Science Foundation of China (No.81773437).

## Conflict of Interest

The authors declare that the research was conducted in the absence of any commercial or financial relationships that could be construed as a potential conflict of interest.
