# Supplementary material for: Arachidonic Acid Metabolism Controls Macrophage Alternative Activation Through Regulating Oxidative Phosphorylation in PPARγ Dependent Manner
Source: Front Immunol. 2021 Jun 3;12:618501. doi: 10.3389/fimmu.2021.618501 (PMC8211451; doi:10.3389/fimmu.2021.618501)
Supplement: Supplementary file 1 [file DataSheet_1.pdf]

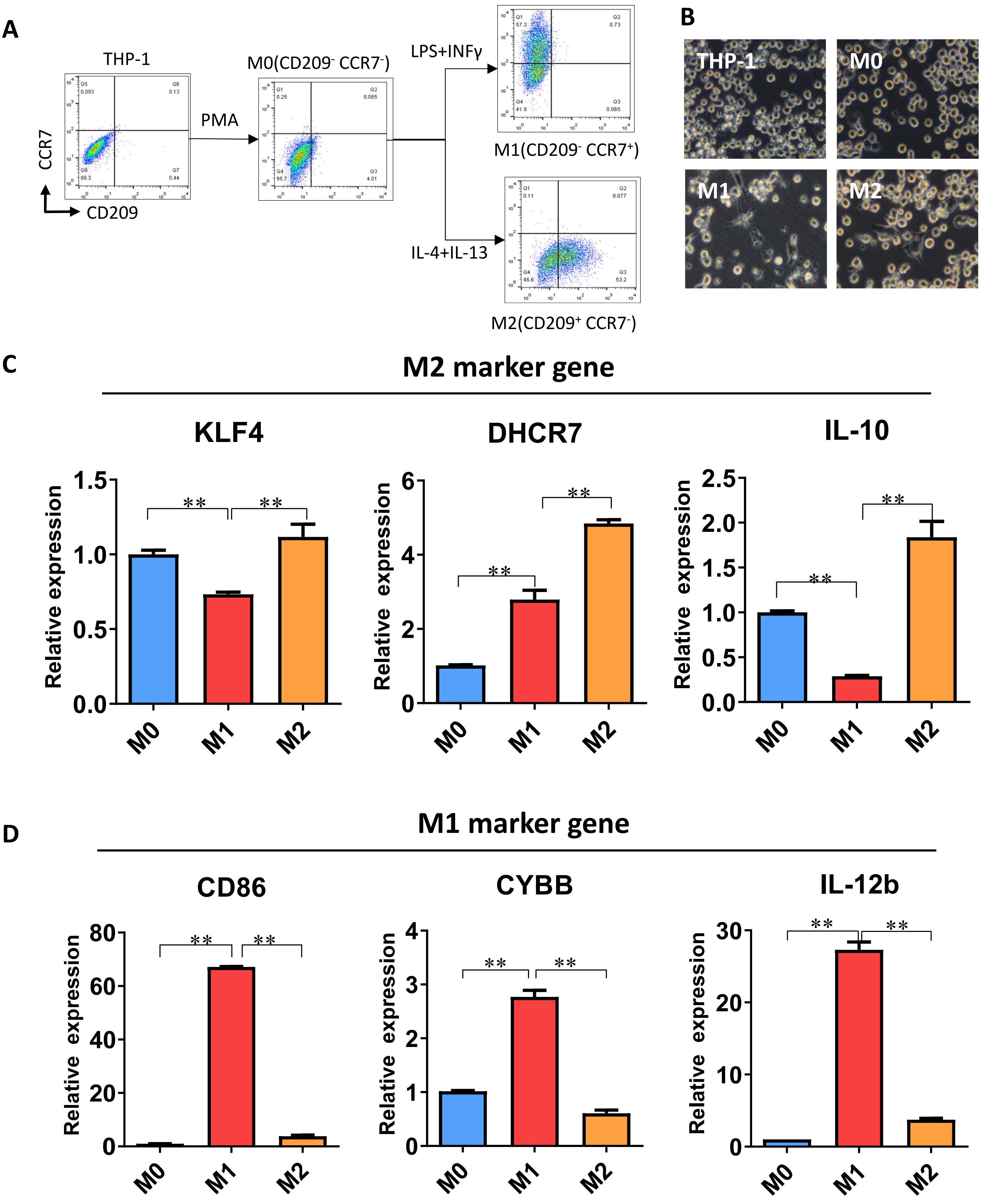

Supplementary Figure S1. Characteristics of THP-1 derived M1/M2. **(A)** Surface marker (CCR7+CD209) detected by flow cytometry distinguished M1/M2. **(B)** Morphology alternation during differentiation and polarization. **(C and D)** qRT-PCR detected M2 or M1 macrophages markers. All data represent mean ± S.D. (n=3). *P< 0.05; ** P < 0.01.

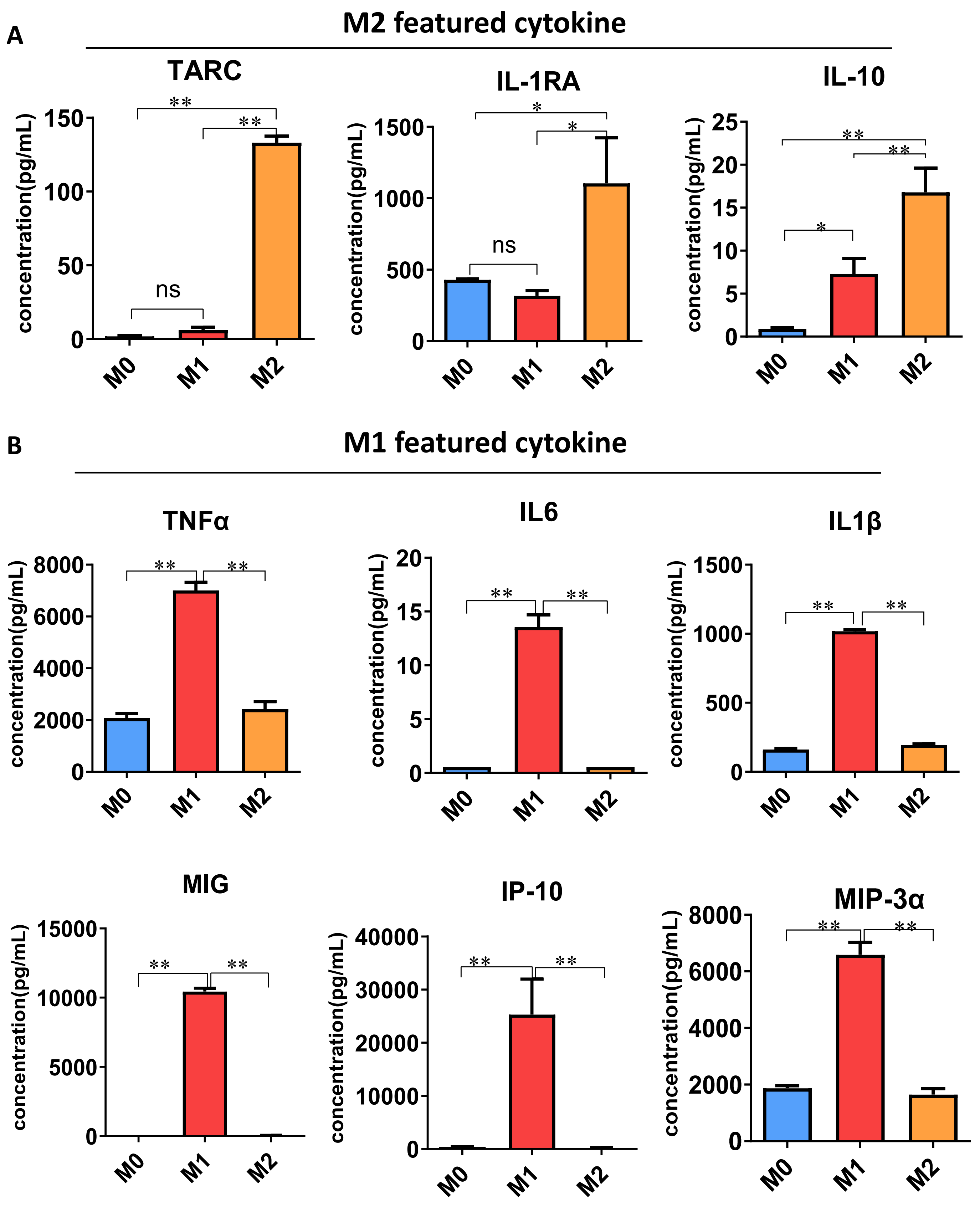

Supplementary Figure S2. Functional cytokines of THP-1 derived M1/M2. **(A and B**) cytokines level of M2 (A) or M1 (B) detected by legendplex panel. All data represent mean ± S.D. (n=3). *P< 0.05; ** P < 0.01; ns, no significance.

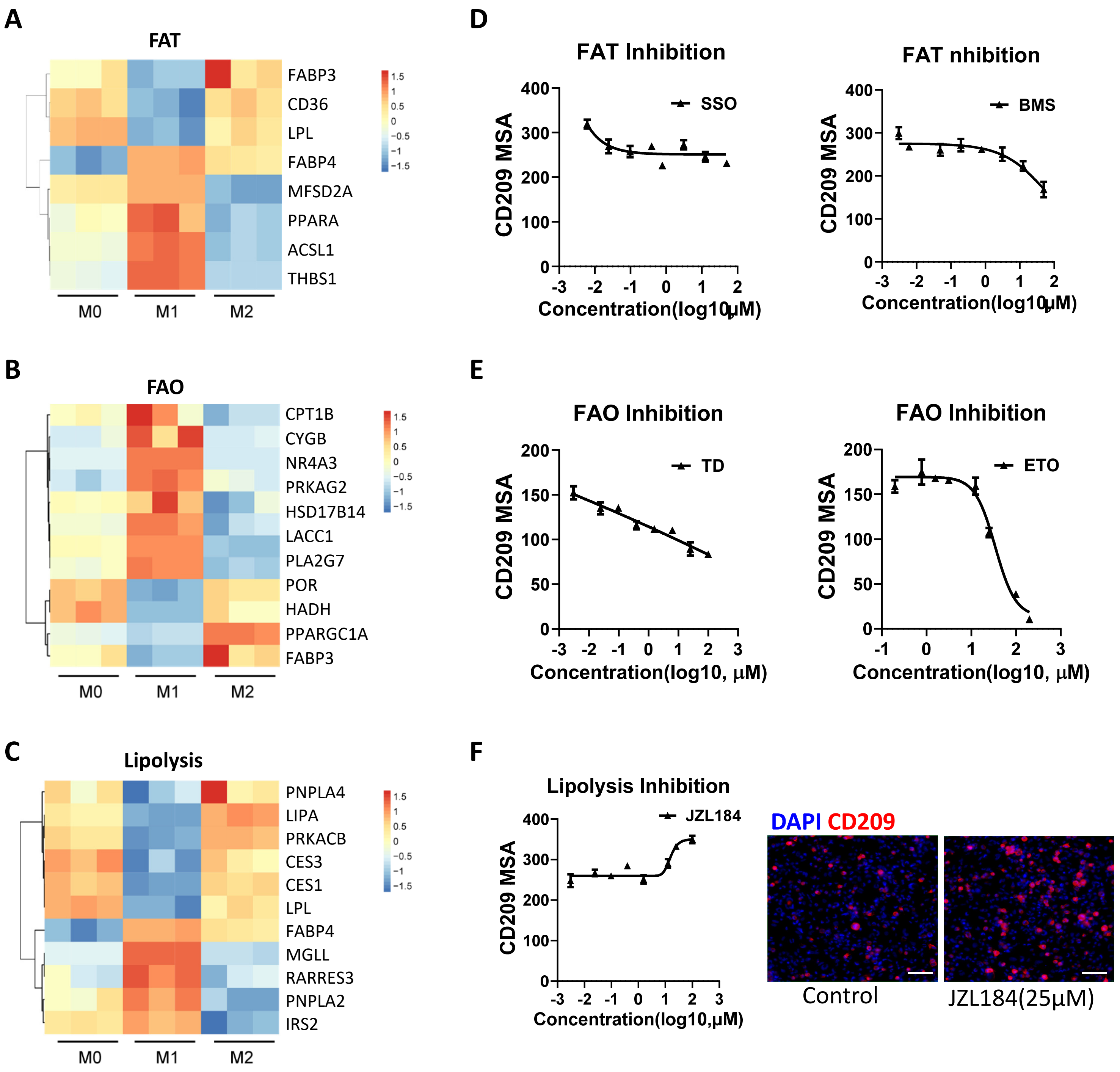

Supplementary Figure S3. Lipid metabolism closely correlates to macrophage polarization. **(A-C)** Heatmaps of differentially expressed gene in THP-1 derived macrophages related to fatty acids transport (FAT), fatty acid oxidation (FAO), lipolysis, respectively. **(D-F)** Dose response curves of CD209 expression in THP-1 derived M2 macrophages with inhibitors treated as indicated. All data represent mean ± S.D. (n=3). MSA. mean stain area. Right panel of (F): representative images, scale bar: 100 μm.

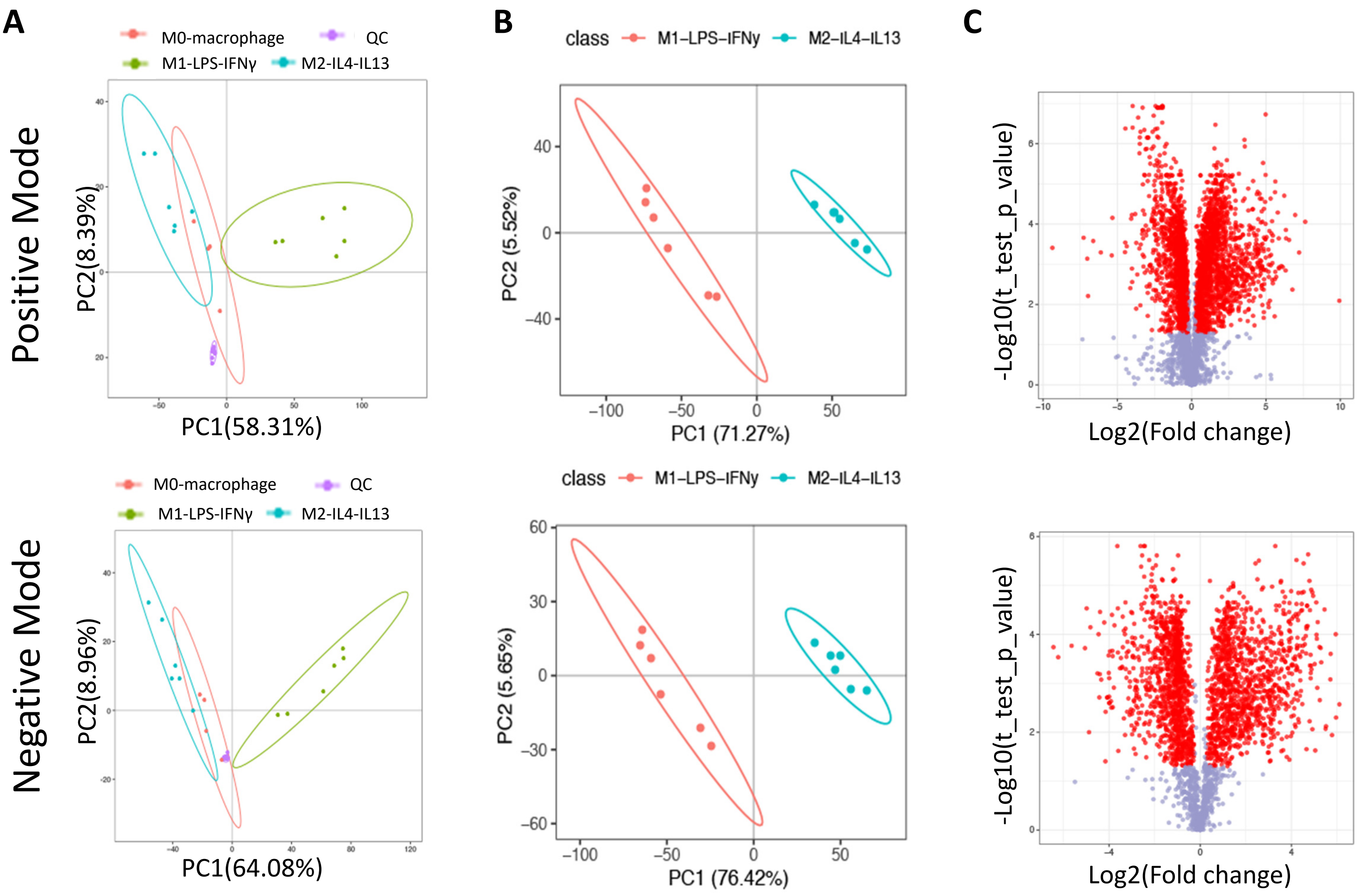

Supplementary Figure S4. Distinct lipidomics characteristics of M1/M2 macrophages. **(A)** Principal components analysis of metabolites from positive or negative ion mode. Quality control samples QC indicated a good quality of the collected data. Each point represents a sample. **(B)** Partial least squares-discriminant analysis revealed distinct metabolites profile of M1/M2 macrophages. **(C)** Overall differential metabolites of M1/M2 macrophages. Fold change ≥ 1.2 or ≤ 0.83 and q-value < 0.05 were set as screening standards. Red: differential metabolites; grey: metabolites without difference.

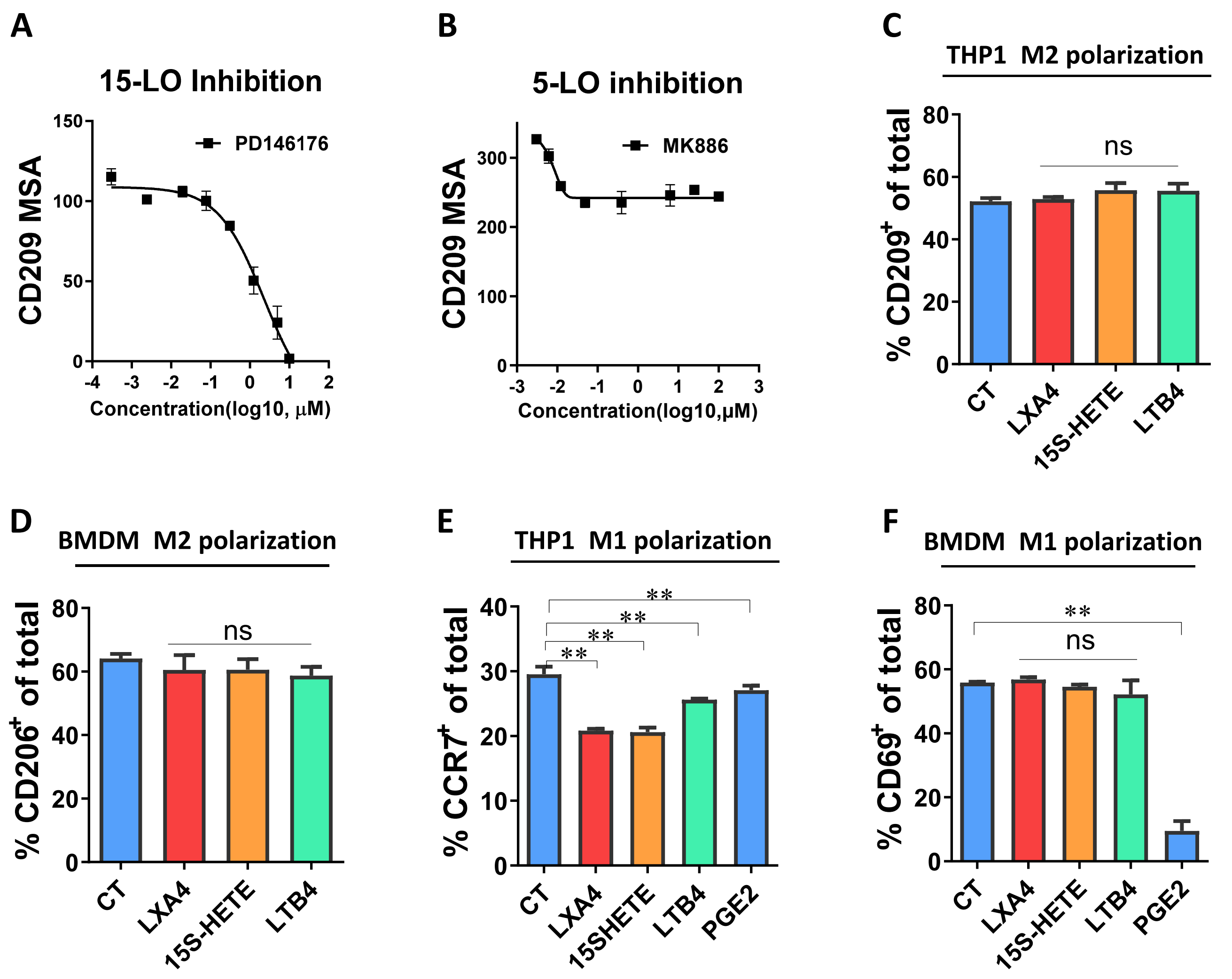

Supplementary Figure S5. Arachidonic acid metabolic enzymes and metabolites regulated macrophages polarization. **(A and B)** Dose response curves for 15-lipoxygenase (15-LO) or 5-lipoxygenase (5-LO) in M2 polarization. **(C and D)** CD209 or CD206 expression in THP-1 or BMDM model. Cells were treated with LXA4 (50 ng/mL), 15S-HETE (50 ng/mL) or LTB4 (100 nM) during M2 polarization. **(E and F)** CCR7 or CD69 expression in THP-1 or BMDM model. Cells were treated with LXA4 (50 ng/mL), 15S-HETE (50 ng/mL), LTB4 (100 nM), PGE2 (2 μM) during M1 polarization. All data represent mean ± S.D. (n=3). *P< 0.05; ** P < 0.01; ns, no significance

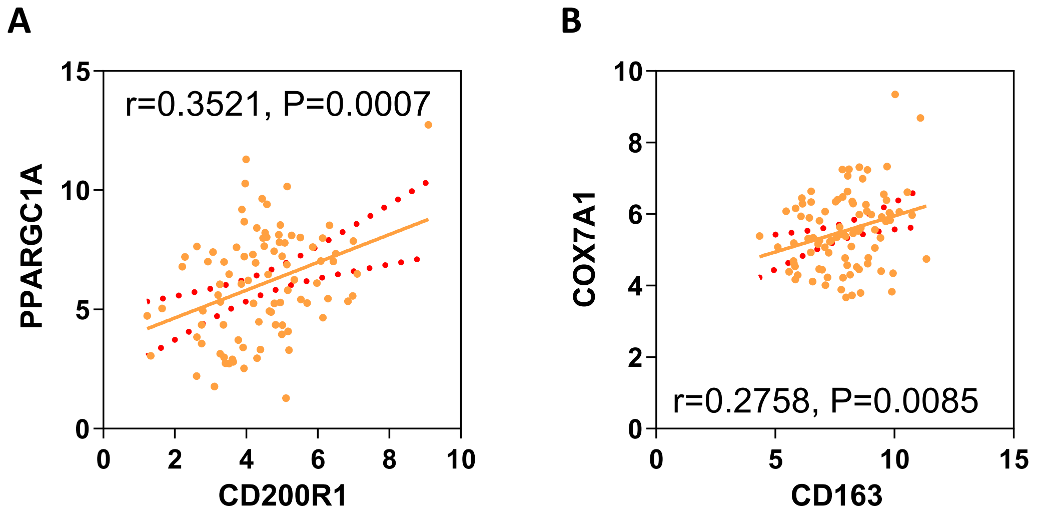

Supplementary Figure S6. Oxidative phosphorylation correlates to M2 TAM in ESCC. **(A and B)** Correlation analysis of oxidative phosphorylation (PPARGC1A, COX7A1) and M2 macrophages (CD200R1, CD163) in human ESCC from TCGA database.

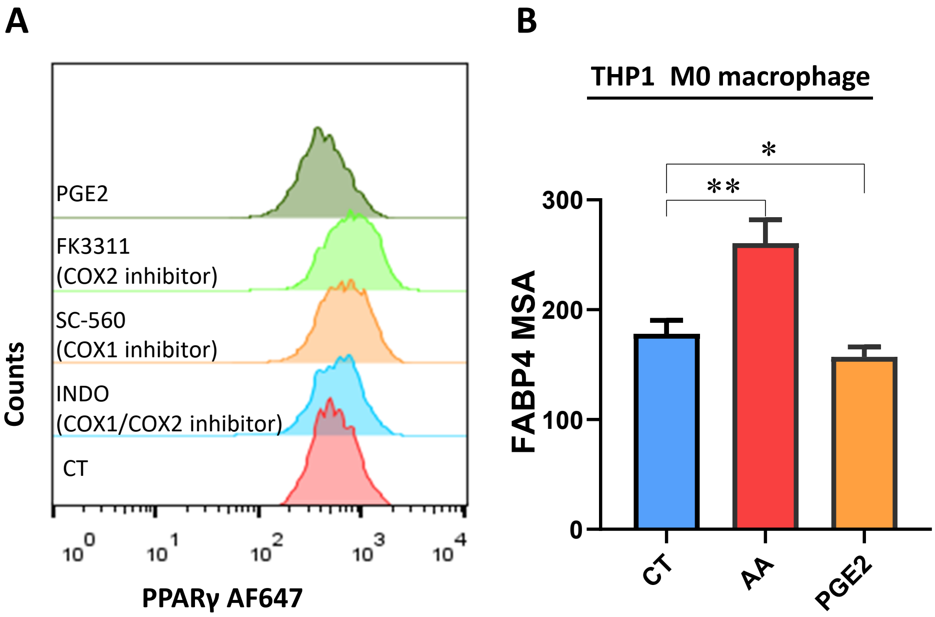

Supplementary Figure S7. Expression and transcription activity of PPARγ were inhibited by PGE2 treatment. **(A)** Representative fluorescence histogram of macrophages PPARγ from flow cytometry with treatments as indicated. **(B)** Expression of macrophages FABP4 (indicator for PPARγ transcription activity) with treatment as indicated. M0 macrophages were treated with PGE2 (2 μM), FK3311 (10 μM), SC-560 (10 μM), Indomethacin (10 μM), AA (50 μM). All data represent mean ± S.D. (n=3). *P< 0.05; ** P < 0.01.

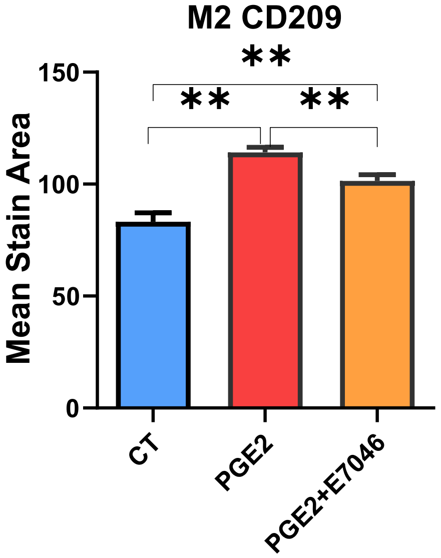

Supplementary Figure S8. Blockade of EP4 did not abolish PGE2-promoted M2 polarization. THP-1 derived macrophages were treated with PGE2 (2 μM) with/without E7046 (10 μM) during M2 polarization. All data represent mean ± S.D. (n=3). *P< 0.05; ** P < 0.01.

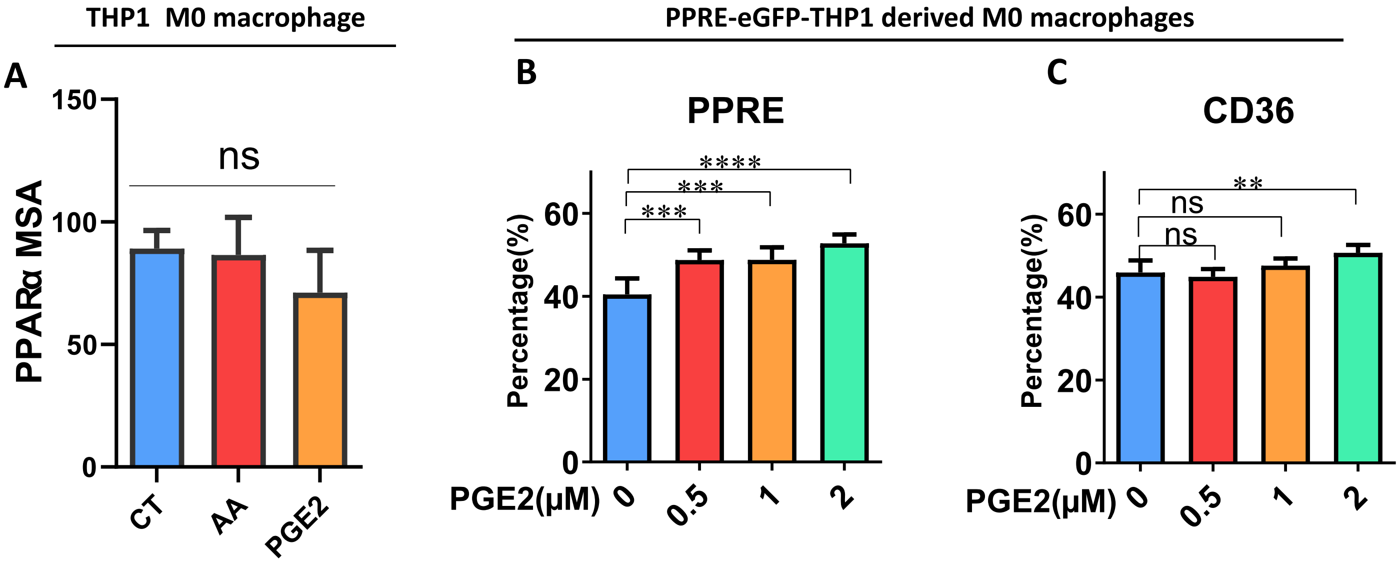

Supplementary Figure S9. The involvement of PPARs in AA/PGE2 mediated M2 polarization. **(A)** Protein expression of PPARα in macrophages treated with AA (50 μM) or PGE2 (2 μM) for 48 h. **(B and C)** PPRE activation (B) and CD36 expression (C) in macrophages treated as indicated for 48 h. All data represent mean ± S.D. (n=3). *P< 0.05; ** P < 0.01; ns, no significance.

Table S1 Key resources

REAGENTS OR RESOURCES	SOURCE	IDENTIFIER
**Biological material**		
THP-1	American Type Culture Collection	TIB-202
Bone marrow derived monocytes (wild type)	C57BL/6 mice	/
Bone marrow derived monocytes (PPARG KO)	Jackson Labs	/
**Chemicals, Antibodies, cytokines**		
Anti-CCR7	Biolegend	Cat#353204
Anti-CD209	Biolegend	330106;330104
Anti-CD206	Biolegend	141706
Anti-CD69	Biolegend	104518
Arachidonic acid (AA)	Sigma Aldrich	A3611
ATP5A	Abcam	ab14748
BMS309403 (BMS)	MedChemExpress	HY-101903
CPT1A	Proteintech	15184-1-AP
Etomoxir sodium salt (ETO)	MedChemExpress	HY-50202A
FASN-IN-4 tosylate (FAI)	MedChemExpress	HY-12648A
FABP4 PE	LSBio	LS-C650165
Human Trustain FcX	Biolegend	422302
Fatostatin (FATO)	MedChemExpress	HY-14452
FK 3311	MedChemExpress	HY-14445
FT113 (FT)	MedChemExpress	HY-111551
Human IL-4	Peprotech	200-04
Human IL-13	Peprotech	AF-200-13
Human IFNγ	Peprotech	300-02
IACS-10759 (IA)	Selleck	S8731
Indomethacin (INDO)	MedChemExpress	HY-14397
JZL184	MedChemExpress	HY-15249
Lipopolysaccharides (LPS)	Sigma Aldrich	L6529
Murine M-CSF	Peprotech	315-02
Murine IL-4	Peprotech	214-14
Murine IL-13	Peprotech	210-13
Murine IFNγ	Peprotech	315-05
3-Nitropropionic acid (NP)	Selleck	S3652
PPARgamma	Cell signaling technology	2435S
Prostaglandin E2 (PGE2)	Sigma Aldrich	P0409
Phorbol 12-myristate 13-acetate (PMA)	Sigma Aldrich	P8139

Sulfosuccinimidyl oleate(SSO)	Sigma Aldrich	SML2148
Trimetazidine (TD)	MedChemExpress	HY-B0968A
VLX600 (VLX)	MedChemExpress	HY-12406
**Critical Commercial assays**		
Foxp3 / Transcription Factor Staining Buffer Set	Invitrogen	00-5523-00
LEGENDplex Human Macrophage/Microglia Panel	biolegend	740502
RNeasy Mini Kit	QIAGEN	74104
**Deposited Data**		
RNA-sequencing data	This paper	GSE159112, GSE159120; GSE134067
**Software and Algorithms**		
Gene Set Enrichment Analysis (GSEA) (v4.0.3)	[2]	http://software.broadinstitute.org/gsea/index.jsp
Metabolite Set Enrichment Analysis (MSEA)(v4.0)	[3]	https://www.metaboanalyst.ca/
Joint Pathway analysis	[3]	https://www.metaboanalyst.ca/
GraphPad prism (v 6.01)	GraphPad Software	N/A
ImageXpress	Molecular Device	
**Other**		
DMEM	Gibco	11995065
Fetal Bovine Serum (FBS)	Gibco	10099141C
Fixation and Permeabilization Solution	BD Bioscience	554722
FluoroBrite ™ DMEM	Gibco	A1896702
Hoechst 33342	Solarbio	C0031
penicillin-streptomycin	Gibco	15140122
Perm/Wash	BD Bioscience	554723
RPMI 1640 (ATCC modification)	Gibco	A1049101
2-mercaptoethanol	Sigma Aldrich	M3148

Supplementary Table S2

Arachidonic acid metabolism associated metabolites from lipidomics analysis

Mode	Ratio	Label	Description
negative	4.06	6.58_397.2261m/z	Prostaglandins(PGH2;PGE2;PGD2;PGI2);TXA2;Lipoxin A4;Lipoxin B4;20-hydroxy LTB4;15-keto-PGF2alpha
positive	2.82	0.96_372.2761m/z	11beta-PGF2;15-F2t-IsoP;PGF2alpha;11,12,15-THETA;Trioxilin A3;11,14,15-THETA
positive	2	1.43_586.3112m/z	LTF4
negative	1.76	3.04_411.2041m/z	20-carboxy-LTB4
positive	1.29	3.05_327.2306m/z	Arachidonic acid
positive	0.78	1.12_338.2474n	5,6-DHET;8,9-DiHETrE;11,12-DiHETrE;14,15-DiHETrE
positive	0.75	0.76_334.2349m/z	15-Deoxy-d-12,14-PGJ2
positive	0.72	1.13_317.2101m/z	12-oxo-LTB4;PGA2;PGB2;PGC2;PGJ2;delta-12-PGJ2;5,6-Ep-15S-HETE
positive	0.65	2.87_336.2517m/z	LTA4;15-KETE;5-KETE;12-KETE
negative	0.41	0.68_355.2036m/z	15S-HETE;5S-HETE;20-HETE;19S-HETE;5,6-EET;(+/-)8,9-EpETrE;(+/-)11,12-EpETrE;(+/-)14,15-EpETrE;8S-HETE;12S-HETE;16R-HETE;9S-HETE;11R-HETE;12R-HETE;8R-HETE

Supplementary Table S3

Detail information for top 20 GSEA enrichment pathways in PGE2 treated M2 polarization.

NAME	SIZE	ES	NES	NOM p	FDR q-
STAPHYLOCOCCUS_AUREUS_INFECTION_-_HOMO_SAPIENS_(HUMAN)(HSA05150)	301	0.66548	1.40566	0	0.738042
ANTIGEN_PROCESSING_AND_PRESENTATION_-_HOMO_SAPIENS_(HUMAN)(HSA04612)	211	0.722176	1.392314	0	0.570246
IL-17_SIGNALING_PATHWAY_-_HOMO_SAPIENS_(HUMAN)(HSA04657)	140	0.510845	1.376146	0	0.489651
PHAGOSOME_-_HOMO_SAPIENS_(HUMAN)(HSA04145)	453	0.509968	1.357369	0	0.465207
OSTEOCLAST_DIFFERENTIATION_-_HOMO_SAPIENS_(HUMAN)(HSA04380)	191	0.461932	1.343462	0	0.443978
HEMATOPOIETIC_CELL_LINEAGE_-_HOMO_SAPIENS_(HUMAN)(HSA04640)	199	0.655428	1.341753	0	0.386636
TH17_CELL_DIFFERENTIATION_-_HOMO_SAPIENS_(HUMAN)(HSA04659)	166	0.533104	1.338639	0	0.338688
GLYCOSAMINOGLYCAN_BIOSYNTHESIS_-_CHONDROITIN_SULFATE_/_DERMATAN_SULFATE_-_HOMO_SAPIENS_(HUMAN)(HSA00532)	28	0.601301	1.332624	0.181818	0.317932
TUBERCULOSIS_-_HOMO_SAPIENS_(HUMAN)(HSA05152)	398	0.540087	1.332295	0	0.288273
LEISHMANIASIS_-_HOMO_SAPIENS_(HUMAN)(HSA05140)	242	0.591083	1.328794	0	0.267774
OXIDATIVE_PHOSPHORYLATION_-_HOMO_SAPIENS_(HUMAN)(HSA00190)	861	0.543396	1.32836	0	0.248068
INFLAMMATORY_BOWEL_DISEASE_-_HOMO_SAPIENS_(HUMAN)(HSA05321)	100	0.584769	1.326967	0	0.231645
VARIOUS_TYPES_OF_N-GLYCAN_BIOSYNTHESIS_-_HOMO_SAPIENS_(HUMAN)(HSA00513)	66	0.573851	1.310889	0	0.240281
GRAFT-VERSUS-HOST_DISEASE_-_HOMO_SAPIENS_(HUMAN)(HSA05332)	99	0.752296	1.30874	0	0.226761
TYPE_I_DIABETES_MELLITUS_-_HOMO_SAPIENS_(HUMAN)(HSA04940)	123	0.665351	1.305426	0	0.230762
NON-ALCOHOLIC_FATTY_LIVER_DISEASE_-_HOMO_SAPIENS_(HUMAN)(HSA04932)	626	0.494478	1.305105	0	0.219527
TYROSINE_METABOLISM_-_HOMO_SAPIENS_(HUMAN)(HSA00350)	67	0.48297	1.299975	0	0.227472
VIRAL_MYOCARDITIS_-_HOMO_SAPIENS_(HUMAN)(HSA05416)	276	0.578541	1.298735	0	0.217668
ESTROGEN_SIGNALING_PATHWAY_-_HOMO_SAPIENS_(HUMAN)(HSA04915)	322	0.459586	1.290178	0	0.222081
RHEUMATOID_ARTHRITIS_-_HOMO_SAPIENS_(HUMAN)(HSA05323)	209	0.583726	1.287354	0	0.217427
